# Agreement of in vitro orthodontic measurements on dental plaster casts and digital models using Maestro 3D ortho studio software

**DOI:** 10.1002/cre2.605

**Published:** 2022-06-19

**Authors:** Elaheh Rafiei, Alireza Haerian, Pooya Fadaei Tehrani, Mohammad Shokrollahi

**Affiliations:** ^1^ Faculty of Dentistry University of British Columbia Vancouver British Columbia Canada; ^2^ Dental Students Research Center Shahid Sadoughi University of Medical Sciences Yazd Iran

**Keywords:** dental model, orthodontics, plaster casts, software, three‐dimensional imaging

## Abstract

**Objective:**

Diagnostic casts are one of the standard components of orthodontic records. But they have several drawbacks such as the need for physical space for storage and the risk of breaking due to their brittle composition. Today, the digitalization of orthodontic models is a progress in orthodontics. The purpose of this study was to compare and evaluate common orthodontic linear measurements on plaster casts and digital 3D models using Maestro 3D ortho studio® scanner and software (AGE Solutions®, Pontedera, Italy).

**Materials and Methods:**

Study casts of 30 orthodontic patients were selected. Tooth width, space analysis, Bolton analysis, overjet, overbite, and linear measurements of dental arch dimensions were performed by two examiners on plaster casts and digital models.

**Statistical Analysis:**

Intra‐ and interexaminer agreements were evaluated in both manual and digital methods and paired *t* test was used for evaluating the agreement between the manual and digital measurement. The significance level was set at 0.05.

**Results:**

The intraexaminer agreement was excellent (ICC > 0.75) for most variables in both manual and digital methods. The correlation between the two examiners was significant (*p *< .05) for most manual and digital measurements. The differences between the manual and digital measurements, although maybe statistically significant, were not clinically significant for most variables.

**Conclusion:**

The use of “Maestro 3D” (AGE Solutions, Pontedera, Italy) scanner and software was acceptable for orthodontic diagnostic measurements instead of study casts.

## 1 INTRODUCTION

Diagnosis in orthodontics is a critical element in explaining the correct goals of treatment (Jiménez‐Gayosso et al., 2018). Diagnostic casts are one of the standard components of orthodontic records and play an essential role in diagnosis, patient presentation, treatment planning, evaluating treatment progress, and maintaining records. Tooth size, crowding, spacing, overjet, overbite, and Bolton analysis are routinely measured manually on models (El‐Zanaty et al., 2010). Direct measurements on plaster casts using calipers have been accepted as a clinical standard, but there are several drawbacks to plaster casts such as the need for physical space for their storage and the risk of breaking due to their brittle composition (Fleming et al., 2011; Lippold et al., 2015; Wan Hassan et al., 2016). Today, digitizing orthodontic dental models is a breakthrough in orthodontics, and these virtual models have several benefits such as improved efficiency, rapid retrieval of digital information from patient records, a quick exchange of patient data for consultation and referral, cost savings, no need for physical space, elimination of the risk of damage or cracking, and ease of digital measurement (Wiranto et al., 2013). So far, many studies have been conducted to compare the analysis of digital models with the gold standard method (caliper measurement of plaster models) (Camardella et al., 2017; Czarnota et al., 2016; Hassan et al. 2016; Reuschl et al., 2016). In a study conducted by Jiménez‐Gayosso et al. (2018) to evaluate the accuracy of the Maestro3D Ortho Studio scanner, they concluded that the manual and digital measurement models were similar for both vertical and transverse measurements. Another study by Grünheid et al. (2014) examined the accuracy, reproducibility, and timing of dental measurements using three different digital models (emodels, SureSmile models, Anatomodels). The results indicated that tooth width measurements on digital models can be as accurate as plaster casts and even more repeatable and faster. Some studies found a statistically significant difference and concluded that measurements on digital models were significantly larger (Asquith et al., 2007; Goonewardene et al., 2008; Naidu & Freer, 2013; Sousa et al., 2012; Stevens et al., 2006), whereas in other reviews the values measured on digital models were substantially smaller (Abizadeh et al., 2012; Mullen et al., 2007; Watanabe‐Kanno et al., 2009), so it may be understood that there is no agreement on the dimensional accuracy of digital models made by plaster cast scans. Due to the contradictory results obtained in the previous research and the limited amount of research on the accuracy of 3D scanners and orthodontic software produced by the same manufacturer and also because previous study (Martin et al., 2015) has shown that the Maestro 3D (AGE Solutions®, Pontedera, Italy) scanner has the highest accuracy compared to many other commercially available desktop orthodontic scanners, the purpose of this study was to compare and evaluate in vitro conventional orthodontic linear measurements on plaster casts and 3D digital models using the scanner and software of Maestro 3D ortho studio® (AGE Solutions®, Pontedera, Italy).

## 2 MATERIALS AND METHODS

**Figure 1:**
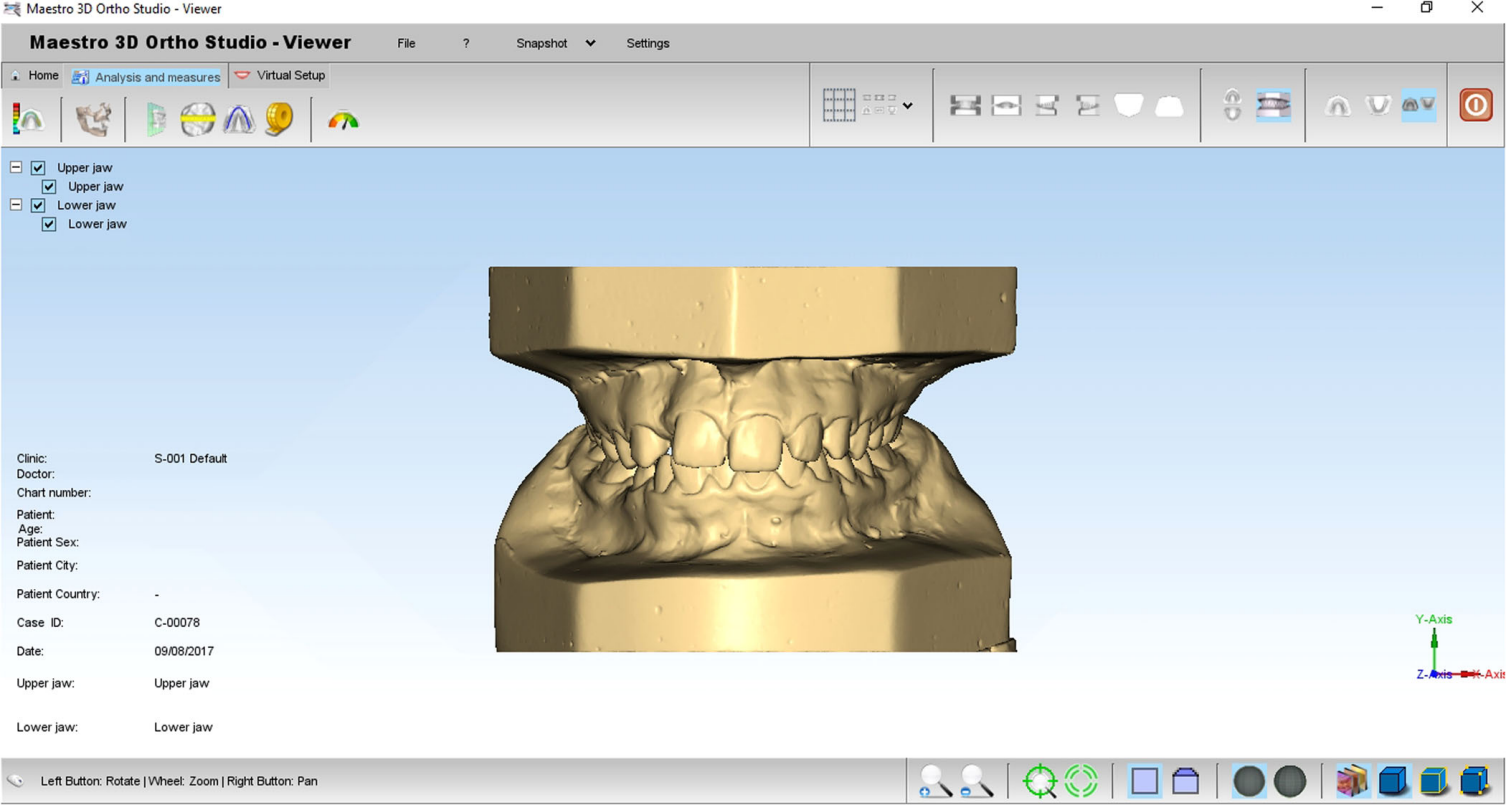
An overview of the Maestro 3D ortho studio® (AGE Solutions®, Pontedera, Italy) environment.

This study was reviewed and approved by the Research Ethics Committee. A study cast of 30 orthodontic patients referred to the Department of Orthodontics was selected. Inclusion criteria for the study included: fully erupted permanent dentition (first molar to one side to the other in both jaws), no previous orthodontic treatment, no structural and morphological abnormalities, no extensive restorations that extend to the proximal surface, with no loss of mesiodistal or labiolingual dimension due to caries, repair or fracture and exclusion criteria included: casts that were excluded for any reason, such as inadequate scanning or artifacts. Tooth width, space analysis, Bolton analysis, overjet, overbite, and linear measurements of dental arch dimensions were performed by two examiners on plaster casts using two methods:

1. Traditional measurement by digital caliper “Guang Lu, China” with measurement precision of 0.01 mm, which used no magnification to measure plaster models manually.
2. Measurement of digital models by Maestro 3D ortho studio® software (AGE Solutions®, Pontedera, Italy), version 4 with 0.01 mm accuracy, which has the zoom and rotation capabilities when analyzing digital models (Figure 1).

First, all 30 pairs of plaster casts were scanned by the digital optical scanner “Maestro 3D (AGE Solutions, Pontedera, Italy)” with a precision of 10 μm; each pair of casts was scanned three times. The first scan of both models was performed to record their relationship, the second time the mandibular casts were scanned separately, and the third time, the maxillary cast was scanned independently. After that, various steps of digital file preparation were performed by Maestro 3D ortho studio® software (AGE Solutions®, Pontedera, Italy), version 4. Before the measurement of the desired parameters, three samples were evaluated jointly by two examiners and the choice of points on both plaster and digital models was discussed to calibrate the examiners and decrease the amount of interexaminer variability. This joint evaluation was performed on both digital and plaster models. At first, the variables in both study methods were determined by the first examiner, who was trained and calibrated. The measurements were performed by two examiners (Inter Examiner Agreement) for 15 plaster and digital models to increase the accuracy of the research and the reliability of the results. The first examiner also performed the measurements on the 15 plaster and digital models after 2 weeks (Intra Examiner Agreement). After completion of manual and digital measurements, data were entered into SPSS software (v. 23; IBM, NY, USA). Then statistical analysis was done by the mentioned software. Intraexaminer agreement for both manual and digital methods was evaluated separately by statistical analysis of interclass correlation coefficients (ICC). Pearson correlation statistical analysis was performed to evaluate interexaminer agreement in both manual and digital measurement methods. A paired *t* test was used for the statistical analysis of the two methods. The significance level was set at 0.05.

## 3 RESULTS

### 3.1 Intraexaminer agreement

ICC of jaw measurements (single measurements) averaged 0.90 ± 0.02 for manual methods and 0.86 ± 0.03 for digital models. The intraexaminer agreement for all measurements in the manual method was excellent (ICC > 0.75) (Tables 1A and 1B), except for the parameters: width 41 (ICC = 0.175), width 16 (ICC = 0.592), anterior Bolton ratio (ICC = 0.70), total Bolton ratio (ICC = 0.688) which are marked with (^a^) in the table. Agreement in these parameters was insufficient, moderate to good, moderate to good, and moderate to good, respectively. The intraexaminer agreement for digital measurement models were excellent for all parameters (ICC > 0.75) (Tables 1A and 1B), except for the parameters width 32 (ICC = 0.612), width 34 (ICC = 0.418), width 45 (ICC = 0.301), width 46 (ICC = 0.009) specified in the table marked with (^a^). Agreement in these parameters was insufficient, moderate to good, moderate to good, and moderate to good, respectively. The ICC value in the manual measurement method (plaster casts) was at its lowest for tooth width 41 (ICC = 0.175), and on the digital models for tooth width 46 (ICC = 0.009).

**Table 1A:** Intraexaminer agreement on the manual method (plaster casts) and digital Maestro™ models: Mesiodistal width of teeth—interclass correlation coefficient test

	Manual method (plaster casts)			Maestro™ digital models		
Variable	ICC	95% CI	*p* value	ICC	CI 95%	*p* value
T‐W 11	0.977	0.932–0.992	<.0001	0.997	0.932–0.992	<.0001
T‐W 12	0.959	0.884–0.986	<.0001	0.904	0.738–0.967	<.0001
T‐W 13	0.938	0.827–0.979	<.0001	0.914	0.764–0.970	<.0001
T‐W 14	0.861	0.637–0.951	<.0001	0.938	0.825–0.979	<.0001
T‐W 15	0.957	0.876–0.985	<.0001	0.930	0.806–0.976	<.0001
T‐W 16	0.59^a^	0.134–0.842	.008	0.928	0.800–0.975	<.0001
T‐W 21	0.996	0.989–0.999	<.0001	0.979	0.940–0.993	<.0001
T‐W 22	0.994	0.982–0.998	<.0001	0.958	0.880–0.986	<.0001
T‐W 23	0.994	0.982–0.998	<.0001	0.967	0.904–0.989	<.0001
T‐W 24	0.976	0.930–0.992	<.0001	0.955	0.871–0.985	<.0001
T‐W 25	0.941	0.833–0.980	<.0001	0.954	0.870–0.984	<.0001
T‐W 26	0.996	0.901–0.988	<.0001	0.885	0.692–0.690	<.0001
T‐W 31	0.990	0.971–0.997	<.0001	0.785	0.472–0.922	<.0001
T‐W 32	0.974	0.925–0.991	<.0001	0.612^a^	0.166–0.851	.006
T‐W 33	0.786	0.475–0.923	<.0001	0.840	0.588–0.943	<.0001
T‐W 34	0.891	0.707–0.962	<.0001	0.418^a^	−0/100 – 0.758	0.054
T‐W 35	0.917	0.772–0.971	<.0001	0.904	0.738–0.967	<.0001
T‐W 36	0.908	0.748–0.968	<.0001	0.982	0.948–0.994	<.0001
T‐W 41	0.175^a^	−0.353–0.619	.258	0.880	0.682–0.958	.002
T‐W 42	0.954	0.869–0.984	<.0001	0.893	0.713–0.963	<.0001
T‐W 43	0.834	0.575–0.941	<.0001	0.866	0.647–0.953	<.0001
T‐W 44	0.969	0.909–0.989	<.0001	0.797	0.497–0.927	<.0001
T‐W 45	0.941	0.833–0.980	.005	0.301^a^	−0.231–0.694	.129
T‐W 46	0.907	0.747–0.968	<.0001	0.009^a^	−0.490–0.504	.487

Abbreviations: CI, confidence interval; ICC, interclass correlation; T‐W, tooth width.

^a^ ICC < 0.75.

**Table 1B:** Intraexaminer agreement on the manual method (plaster casts) and digital models of Maestro™: Linear parameters—interclass correlation coefficient test

	Manual method (plaster casts)			Maestro™ digital models		
Variable	ICC	CI 95%	*p* value	ICC	CI 95%	*p* value
Max. Space available	0.998	0.995–0.999	<.0001	0.994	0.981–0.998	<.0001
Man. Space available	0.991	0.974–0.997	<.0001	0.991	0.973–0.997	<.0001
Max. Space required	0.991	0.975–0.997	<.0001	0.991	0.974–0.997	<.0001
Man. Space required	0.983	0.951–0.994	<.0001	0.948	0.52–0.982	<.0001
Anterior Bolton ratio	0.703^a^	0.316–0.889	.001	0.783	0.468–0.922	<.0001
Total Bolton ratio	0.688^a^	0.289–0.883	.002	0.843	0.595–0.944	<.0001
Max. IMW	0.999	0.998–1.000	<.0001	0.998	0.995–0.999	<.0001
Man. IMW	0.998	0.994–0.999	<.0001	0.987	0.962–0.996	<.0001
Max. ICW	0.998	0.995–0.999	<.0001	0.996	0.989–0.999	<.0001
Man. ICW	0.998	0.995–0.999	<.0001	0.998	0.994–0.999	<.0001
Overjet	0.995	0.986–0.998	<.0001	0.940	0.832–0.979	<.0001
Overbite	0.999	0.998–1.000	<.0001	0.985	0.957–0.995	<.0001

Abbreviations: CI, confidence interval; ICC, interclass correlation; ICW, intercanine width; IMW, intermolar width; Man, mandibular; Max, maxillary.

^a^ ICC < 0.75.

### 3.2 Interexaminer agreement

The interexaminer agreement showed that the correlation between the two examiners in the manual method was significant for all variables (*p* < .05) (Tables 2A and 2B) except for tooth width 46 (*p* = .23), and in the digital method (3D models) the correlation between two examiners was significant for all variables (Tables 2A and 2B) except for tooth width 45 (*p* = .174) and tooth width 46 (*p* = .561) marked by (^a^) in the table.

**Table 2A:** Interexaminer agreement on the manual method (plaster casts) and digital Maestro™ models: Mesiodistal width of teeth—Pearson correlation test

	Manual method (plaster casts)		Maestro™ digital models	
Variable	Pearson correlation	*p* value	Pearson correlation	*p* value
T‐ W 11	0.897	<.0001	0.946	<.0001
T‐ W 12	0.926	<.0001	0.897	<.0001
T‐ W 13	0.926	<.0001	0.925	<.0001
T‐ W 14	0.762	0.001	0.925	<.0001
T‐ W 15	0.888	<.0001	0.848	<.0001
T‐ W 16	0.727	.002	0.908	<.0001
T‐ W 21	0.946	<.0001	0.934	<.0001
T‐ W 22	0.905	<.0001	0.927	<.0001
T‐ W 23	0.889	<.0001	0.901	<.0001
T‐ W 24	0.969	<.0001	0.880	<.0001
T‐ W 25	0.913	<.0001	0.876	<.0001
T‐ W 26	0.891	<.0001	0.948	<.0001
T‐ W 31	0.816	<.0001	0.863	<.0001
T‐ W 32	0.899	<.0001	0.690	.004
T‐ W 33	0.903	<.0001	0.922	<.0001
T‐ W 34	0.947	<.0001	0.951	<.0001
T‐ W 35	0.949	<.0001	0.918	<.0001
T‐ W 36	0.858	<.0001	0.935	<.0001
T‐ W 41	0.896	<.0001	0.899	<.0001
T‐ W 42	0.858	<.0001	0.902	<.0001
T‐ W 43	0.911	<.0001	0.899	<.0001
T‐ W 44	0.954	<.0001	0.888	<.0001
T‐ W 45	0.772	.001	0.371^a^	.174
T‐ W 46	0.854^a^	.23	0.163^a^	.561

Abbreviation: T‐W, tooth width.

^a^ Statistically insignificant correlations.

**Table 2B:** Interexaminer agreement on the manual method (plaster casts) and digital Maestro™ models: Linear parameters—Pearson correlation test

Variable	Manual method (plaster casts)		Maestro™ digital models	
	Pearson correlation	*p* value	Pearson correlation	*p* value
Max. Space available	0.982	<.0001	0.953	<.0001
Man. Space available	0.926	<.0001	0.952	<.0001
Max. Space required	0.954	<.0001	0.981	<.0001
Man. Space required	0.951	<.0001	0.956	<.0001
Anterior Bolton ratio	0.700	.004	0.658	.008
Total Bolton ratio	0.609	.016	0.750	.001
Max. IMW	0.995	<.0001	0.995	<.0001
Man. IMW	0.987	<.0001	0.978	<.0001
Max. ICW	0.991	<.0001	0.995	<.0001
Man. ICW	0.966	<.0001	0.960	<.0001
Overjet	0.946	<.0001	0.936	<.0001
Overbite	0.994	<.0001	0.963	<.0001

Abbreviations: ICW, intercanine width; IMW, intermolar width; Man, Mandibular; Max, Maxillary.

### 3.3 Agreement between measurements made manually (plaster casts) compared to Maestro® digital models

For some of the measured variables, *p* < .05 indicated a statistically significant difference between the manual and digital methods (Tables 3A and 3B). But according to the American Board of Orthodontics Objective Grading System (ABO OGS), a difference of less than 0.5 mm in the vertical, horizontal, and anterior−posterior measurements is not clinically significant (Torassian et al., 2010). Hence, mean differences of less than 0.5 mm in the measurement of tooth width and linear parameters between the two methods, although may be statistically significant, are not clinically significant. The mean difference between total Bolton ratios and anterior Bolton ratios in manual and digital methods were 0.67% and 1.03%, respectively, and these values were converted to millimeters (mm) to assess their clinical relevance (Naidu & Freer, 2013). Using Bolton's formula and mean tooth width in the whole specimen, 0.67% difference in overall Bolton ratios was 0.73 mm in the tooth size and the difference (discrepancy) of 1.03% in the anterior Bolton ratios was 1.32 mm in the tooth size, which is not clinically significant according to Proffit et al. (2018).

**Table 3A:** Agreement between measurements made manually (plaster casts) compared to Maestro™ digital models: Mesiodistal width of teeth—paired *t *test

Pairs	Mean ± SD	Mean differences	*p* value	Pairs	Mean ± SD	Mean differences	*p* value
M‐W11	8.67 ± 0.62	0.12	.020^a^	M‐W31	5.54 ± 0.34	0.09	.002^a^
D‐W11	8.56 ± 0.64			D‐W31	5.45 ± 0.36		
M‐W12	6.94 ± 0.72	0.22	<.0001^a^	M‐W32	6.02 ± 0.37	−0.02	.597
D‐W12	6.72 ± 0.69			D‐W32	6.00 ± 0.37		
M‐W13	7.80 ± 0.60	0.11	.046^a^	M‐W33	6.77 ± 0.52	0.06	.104
D‐W13	7.70 ± 0.49			D‐W33	6.71 ± 0.45		
M‐W14	6.96 ± 0.52	0.11	.003^a^	M‐W34	7.14 ± 0.54	0.08	.012^a^
D‐W14	7.07 ± 0.51			D‐W34	7.22 ± 0.56		
M‐W15	6.63 ± 0.47	−0.12	.009^a^	M‐W35	7.17 ± 0.53	−0.13	.002^a^
D‐W15	6.75 ± 0.56			D‐W35	7.30 ± 0.54		
M‐W16	10.13 ± 0.87	0.27	.019^a^	M‐W36	10.98 ± 0.59	0.10	.021^a^
D‐W16	10.40 ± 0.67			D‐W36	11.08 ± 0.56		
M‐W21	8.75 ± 0.67	0.12	.001^a^	M‐W41	5.50 ± 0.30	0.03	.030^a^
D‐W21	8.63 ± 0.61			D‐W41	5.47 ± 0.33		
M‐W22	6.97 ± 0.68	−0.18	<.0001^a^	M‐W42	5.98 ± 0.50	0.02	.515
D‐W22	6.79 ± 0.72			D‐W42	6.00 ± 0.45		
M‐W23	7.81 ± 0.46	0.16	<.0001^a^	M‐W43	6.72 ± 0.49	−0.04	.363
D‐W23	7.64 ± 0.46			D‐W43	6.69 ± 0.43		
M‐W24	7.05 ± 0.49	0.05	.051	M‐W44	7.20 ± 0.55	−0.06	.138
D‐W24	7.10 ± 0.48			D‐W44	7.26 ± 0.57		
M‐W25	6.62 ± 0.48	−0.06	.123	M‐W45	6.94 ± 0.52	0.34	.028^a^
D‐W25	6.68 ± 0.47			D‐W45	7.27 ± 0.92		
M‐W26	10.17 ± 0.60	0.09	.088	M‐W46	10.91 ± 0.59	0.02	.919
D‐W26	10.26 ± 0.62			D‐W46	10.90 ± 0.95		

Abbreviations: D‐W, digital width; M‐W, manual width; SD, standard deviation.

^a^ Statistically significant.

**Table 3B:** Agreement between measurements made manually (plaster casts) compared to Maestro™ digital models: Linear parameters—paired *t* test

Pairs	Mean ± SD	Mean differences	*p* value	Pairs	Mean ± SD	Mean differences	*p* value
Manual Max. Space available	74.35 ± 5.40	0.59^a^	.088	Manual Max. IMW	46.52 ± 8.22	−1.55^a^	.309
Digital Max. Space available	74.94 ± 5.42			Digital Max. IMW	48.07 ± 2.65		
Manual Man. Space available	65.02 ± 4.24	−0.16	.396	Manual Man. IMW	42.35 ± 2.58	−0.02	.624
Digital Man. Space available	65.17 ± 4.09			Digital Man. IMW	43.33 ± 2.65		
Manual Max. space required	74.19 ± 4.69	−0.57^a^	<.0001^b^	Manual Max. ICW	33.28 ± 2.94	0.03	.474
Digital Max. space required	73.63 ± 4.46			Digital Max. ICW	33.25 ± 2.88		
Manual Man. space required	64.98 ± 3.73	−0.38	.071	Manual Man. ICW	25.62 ± 2.60	−0.01	.749
Digital Man. space required	65.36 ± 3.81			Digital Man. ICW	25.60 ± 2.57		
Manual Anterior Bolton ratio	77.93 ± 2.65	1.03%	<.0001^b^	Manual Overjet	3.25 ± 2.22	0.07	.615
Digital Anterior Bolton ratio	78.96 ± 6.69			Digital Overjet	3.18 ± 2.27		
Manual Total Bolton ratio	92.02 ± 2.50	0.67%	.003^b^	Manual Overbite	2.98 ± 1.75	−0.11	.074
Digital Total Bolton ratio	92.69 ± 2.18			Digital Overbite	2.87 ± 1.80		

Abbreviations: ICW, intercanine width; IMW, intermolar width; Man, Mandibular; Max, Maxillary; SD, standard deviation.

^a^ Clinically significant.

^b^ Statistically significant.

## 4 DISCUSSION

The present study was performed to evaluate and compare different orthodontic parameters on plaster casts and digital models using Maestro 3D ortho studio® software and Maestro 3D scanner (AGE Solutions, Pontedera, Italy). ICC of jaw measurements in the present study for manual and digital models was excellent according to a study conducted by Roberts and Richmond (1997). Czarnota et al. (2016) reported that intraexaminer reliability for some of the parameters was low. In that study, ICC for digital measurements of the width 41 parameter was at its lowest (ICC = 0.61), and in the manual method, it was at its lowest for the overjet parameter (ICC = 0.82). Mullen et al. (2007) found that intraexaminer agreement was slightly higher when using the manual method (plaster casts) compared to the digital method. They hypothesized that this difference was due to the use of a different version of the software for the second measurement in the digital method. In the present study also intraexaminer reliability, although being excellent in both methods, was slightly higher in the manual method compared to the digital technique. One explanation could be due to the examiner's lower experience in using software for measuring digital models. Another explanation is the better repeatability of the manual method which can be attributed to the possibility of physical landmark identification on the plaster casts. The amount of training and coordination of the two examiners in determining the reference points before their formal measurement can affect interexaminer reliability. Due to the calibration performed to identify landmarks between the two examiners in the present study, this issue was minimized. According to the results of the present study in both manual and digital methods, if the practice and coordination of the reference points are sufficient, regardless of different examiners or time intervals, they are reliable measurement tools. In general, in the present study, we tried to reduce the systematic error in the measurements by performing the calibration process; however, the fatigue caused by a large number of measurements and the low experience of the examiners who measure the variables can still cause measurement errors (Hassan et al., 2016). In the present study, the mean differences between the two methods in measuring tooth widths and linear parameters were less than 0.5 mm except for maxillary space available parameters (0.59 mm), and required space in the maxilla (0.57 mm) and intermolar width in the maxilla (1.55 mm). According to the American Board of Orthodontics Objective Grading System (ABO OGS), a difference of less than 0.5 mm in the vertical, horizontal, and anterior−posterior measurements is not clinically significant. The difference between the required and available maxillary space parameters between the manual and digital methods can be attributed to the physical barrier of plaster casts, which can prevent the teeth from being measured properly due to the crowding of teeth. In other words, adjacent teeth can prevent the caliper tip from being placed correctly during the measurement process, which may compromise the accuracy of the results. However, the clinical difference in the required space parameter in the maxilla can be because this parameter is the result of the sum of several separate parameters, and as a result of the sum of the individual measurements, their measurement error increases and becomes clinically meaningful. The difference between the manual and digital methods in the available and required maxillary space parameters was 0.59 and 0.57 mm, respectively, although clinically significant, but this difference was very close to 0.5 mm. The difference in intermolar width can also be related to the instability in determining the points of interest for measuring intermolar width on the cusp tips of the upper first molar teeth. In general, the accuracy of the digital measurement of dental models depends strongly on the correct determination of the points.

Identifying a three‐dimensional relationship on a two‐dimensional computer monitor is an essential factor when measuring. Stevens et al. (2006) noted that, unlike plaster casts that provide accurate 3D representation, the partial rotation of digital models on the computer screen could rapidly alter operator perception. Therefore, examiners need to rotate the digital models to make sure the landmarks are correctly identified. This action can reduce the efficiency of measurements compared to plaster casts. However, some authors argue that digital models are more efficient for measuring Bolton ratios than plaster models because the software can calculate these ratios automatically after they are measured (Lemos et al., 2015; Quimby et al., 2004; Tomassetti et al., 2001). A Bolton discrepancy of less than 1.5 mm is rarely significant, but larger discrepancies create treatment problems in achieving ideal interdigitation, overjet, and overbite, and must be included in the orthodontic problem list (Proffit et al., 2018). In the present study, considering insignificant clinical differences in the measurement of Bolton ratios between the manual and digital methods, it seems that the ease of calculating the Bolton ratios by software in the digital method can confirm the better performance of this method compared to the manual method. Repeat scanning of plaster casts due to artifacts was one of the executive problems of this study. It is recommended that for evaluating the accuracy of the scanner and software studied, a similar study be conducted in samples with different crowding rates (mild, moderate, and severe).

## 5 CONCLUSION

1. Although the measurements performed by Maestro 3D ortho studio® software (AGE Solutions®, Pontedera, Italy) on 3D digital models scanned by Maestro 3D scanners had some significant differences in some parameters with the measurements made by the gold standard method (i.e., plaster cast measurements by digital calipers), the differences were minimal in most landmarks and were not clinically significant.
2. This study demonstrates that the Maestro LED noncontact 3D optical scanner and the related software, Maestro Ortho Studio® (AGE Solutions®, Pontedera, Italy) are clinically useful. So, it could be an additional option to the systems currently available for producing 3D models.
3. If the examiners have sufficient pre‐training, the Maestro scanner and software are reliable measurement tools and can be a good option for replacing plaster casts in orthodontic diagnostic fields.

## AUTHOR CONTRIBUTIONS

Pooya Fadaei Tehrani contributed to reviewing and editing the manuscript and was a major contributor to the writing. Elaheh Rafiei planned the methodology of the study and administrated the project and was a major contributor to the writing of the manuscript. Alireza Haerian contributed to reviewing and editing the manuscript and also provided the resources. Mohammad Shokrollahi performed the investigation and was a major contributor to the writing of the manuscript. All authors read and approved the final manuscript.

## CONFLICT OF INTEREST

The authors declare no conflict of interest.

## ETHICS STATEMENT

This study was reviewed and approved by the Research Ethics Committee of Shahid Sadoughi University of Medical Sciences under the code of IR. SSU. REC.1396.141.

## Data Availability

The data sets used and analyzed during the current study are available from the corresponding author on reasonable request.
